# Characteristics of the Intestine Extracts and Their Effect on the Crude Collagen Fibers of the Body Wall from Sea Cucumber *Apostichopus japonicus*

**DOI:** 10.3390/biology12050705

**Published:** 2023-05-12

**Authors:** Shi-Qi Xu, Zheng-Yu Zhang, Bin Nie, Yi-Nan Du, Yue Tang, Hai-Tao Wu

**Affiliations:** 1 School of Food Science and Technology, Dalian Polytechnic University, Dalian 116034, China; 2 National Engineering Research Center of Seafood, Dalian 116034, China; 3 Collaborative Innovation Center of Seafood Deep Processing, Dalian 116034, China

**Keywords:** sea cucumber, intestinal extracts, collagen fibers, endogenous enzymes, softening

## Abstract

**Simple Summary:**

Sea cucumbers are very sensitive to the influence of environmental factors, and will vomit their intestines and undergo tissue degradation under certain stimuli. In order to determine the effect of endogenous enzymes in the sea cucumber intestine on the body wall during body wall softening, intestine extracts and crude collagen fibers were prepared, respectively. The endogenous enzymes belonging to serine proteases were identified by gelatin zymography. The rheological property results showed that the addition of sea cucumber intestinal extracts reduced the viscosity of crude collagen fibers from 32.7 Pa·s to 5.3 Pa·s. The serine protease inhibitor phenylmethylsulfonyl fluoride inhibited the activity of intestinal extracts, and maintained the viscoelasticity of crude collagen fibers. In this paper, endogenous enzymes in sea cucumber intestinal extracts were identified as serine endopeptidases, which caused softening of the body wall.

**Abstract:**

Sea cucumbers *Apostichopus japonicus* will vomit their intestines during certain stimulations, and the collagen of the body wall will then be degraded. To define the effect of the sea cucumber intestine extracts on the body wall, the intestinal extracts and crude collagen fibers (CCF) of sea cucumber *A. japonicus* were prepared. According to the gelatin zymography, the type of endogenous enzymes in intestinal extracts were mainly serine endopeptidases with optimal activities at pH 9.0 and 40 °C. According to the rheology results, the viscosity of 3% CCF decreased from 32.7 Pa·s to 5.3 Pa·s by adding intestine extracts. The serine protease inhibitor phenylmethanesulfonyl fluoride inhibited the activity of intestinal extracts and increased the viscosity of collagen fibers to 25.7 Pa·s. The results proved that serine protease in the intestinal extracts participated in the process of body wall softening in sea cucumbers.

## 1. Introduction

The sea cucumber *Apostichopus japonicus* is a common aquatic product. It is particularly favoured in China and other Asian nations due to its excellent nutritional content. Growing sea cucumbers is a significant part of the marine economy. In 2022, China produced more than 250 thousand tons of sea cucumbers. However, some factors such as nutrient deficiency, mechanical stimulation, salt concentration, ultraviolet irradiation, and temperature can seriously affect its quality [1,2,3]. The body wall will undergo softening until it becomes completely liquid. Sea cucumber is so sensitive to environmental changes that even small stimuli can cause it to spit out its intestine [4]. Sea cucumber intestines are rich in digestive enzymes, such as pepsin, trypsin, cathepsin B, highly alkaline protease, *β*-1,3-glucanase and other enzymes [5,6,7,8]. The endogenous enzymes from the intestine are then released and touch the body wall of the sea cucumber, which leads to serious nutritional damage during transportation and storage, resulting in serious economic losses. Therefore, it is essential to investigate the body wall softening caused by endogenous enzymes in sea cucumber intestines in detail [9].

The body wall, which contains a variety of different kinds of collagen and other proteins, is the primary edible part of the sea cucumber [10]. Our previous studies have found that the collagen in the sea cucumber body wall was extremely insoluble, even in SDS and urea solution, so the degradation of collagen cannot be observed by electrophoresis [11]. An important phenomenon of sea cucumber body wall softening is protein degradation, which is primarily caused by several different endogenous enzymes, including alkaline proteinase [7], matrix metalloproteinase [12], and gelatinolytic metalloproteinase [13]. Zhao et al. confirmed that the degradation of sea cucumber muscle layer proteins is related to serine proteases and cysteine proteases [14]. However, the characteristics of endogenous enzymes in the sea cucumber intestine and their effect on the property changes of collagen fibers are still unclear.

Serine proteases (EC 3.4.21) contain a large family of endopeptidases that use serine residues as catalytic sites. They are so abundant that over 700 serine endopeptidases have been identified, accounting for a third of known peptidases [15]. They perform a variety of physiological functions such as digestion, immune response, blood clotting, fibrinolysis and reproduction [16]. For aquatic products, endogenous serine proteases may be present in the muscle tissue or digestive tract, such as myofibril-bound serine endopeptidase, sarcoplasmic serine proteinase, trypsin, etc. [17,18,19]. These endogenous enzymes cause degradation of myofibril proteins or collagenous proteins, leading to deterioration of the texture of the product during processing and storage [20].

In this study, the intestines of sea cucumbers were extracted and the properties of collagen-degrading enzymes in the extract were investigated. The optimal conditions and types of endogenous enzymes in the sea cucumber intestinal extract were determined by gelatin zymography. The intestinal extracts were then applied to crude collagen fibers (CCF) from the sea cucumber body wall to simulate the softening process. The effect of the extract on CCF was studied by a rheological test. This study will provide a theoretical basis for the body wall softening in sea cucumbers, and a new idea for reducing the loss caused by the body wall softening.

## 2. Materials and Methods

### 2.1. Materials and Chemicals

Fresh adult sea cucumbers *A. japonicus*, harvested from the Yellow Sea, were purchased from Dalian Liujiaqiao market. The body mass of sea cucumbers was approximately 120 g and their length was approximately 13 cm. Phenylmethylsulfonyl fluoride (PMSF), E-64, and 1,10-phenanthroline were acquired from Sigma Chemical Co. (St. Louis, MO, USA). Gelatin was procured from Sangon Biotech Co. (Shanghai, China). All other reagents were of analytical grade.

### 2.2. Sample Preparation

The CCF was extracted according to the method of Dong et al. [21] with a slight modification. The body wall of the sea cucumber (100 g) was homogenized in 100 mL deionized water and then centrifuged at 13,600× *g* for 10 min. The precipitate was stirred for 30 min with deionized water (1 L) and centrifuged (13,680× *g*, 10 min). This rinsing process was repeated twice. After centrifugation, the precipitate was treated with 1 L of disaggregating buffer containing 0.1 M pH 8.0 Tris-HCl, 5 mM EDTA and 0.5 M NaCl. After gently stirring for 12 h, the samples were centrifuged at 13,680× *g* for 10 min, washed to neutral with deionized water, and stirred in 0.8 L deionized water for 72 h. After centrifugation at 9500× *g* for 5 min, the supernatant was discarded, and the upper part of the precipitate was collected. The lower part was stirred with 0.5 L deionized water for 48 h, and the extraction process was repeated and combined with the previous upper part. The lyophilized precipitate was stirred with 500 volumes (*w*/*v*) of 0.1 M NaOH for 72 h, the precipitate obtained by centrifuging at 17,300× *g* for 10 min was rinsed with distilled water until neutral, and the final lyophilized product was CCF. Except for special instructions, all procedures were performed at 4 °C. Lyophilized CCF was added to ultrapure water and stirred at 2000 rpm for 12 h to fully swell. A final concentration of 3% CCF was used for subsequent rheological studies.

### 2.3. Preparation of Extracts from Sea Cucumber Intestines

Sea cucumber intestines were rinsed in deionized water to remove all dirt and sand. Then, the intestines (10 g) were minced by a grinder with 20 mL of 20 mM Tris-HCl buffer (pH 9.0) containing 10 mM CaCl_2_ (buffer A) and shaken overnight at 4 °C. After centrifugation at 10,000× *g* for 10 min (4 °C), the supernatant was collected as the extract of sea cucumber intestines. The sea cucumber intestinal extract solution was divided into small portions and stored at −80 °C. It was diluted 100 times for subsequent experiments.

### 2.4. Gelatin Zymography Assay

Gelatin zymography was conducted according to the method of Wang et al. [22], with slight modification using 10% separating gel and 5% stacking gel. The sea cucumber intestinal extract solution was mixed with sample buffer at a ratio of 1:4 (*v*/*v*). The sample buffer was composed of 250 mM Tris-HCl (pH 6.8), 5% SDS, 0.05% bromophenol blue, and 50% glycerol. Then, the mixtures (16 μL) were electrophoresed in SDS-PAGE containing 1 mg/mL gelatin at 4 °C. The electrophoresed gels were washed twice with 2.5% (*v*/*v*) Triton X-100 for 30 min each to remove SDS and then rinsed with deionized water. Subsequently, the gels were incubated at the set temperature for 24 h in Tris-HCl buffers at different pH values containing 5 mM CaCl_2_. The temperature was set at 30, 40, 50, and 60 °C, and the pH was set from 6.0 to 11.0, respectively. Finally, the gels were stained with Coomassie bright blue R-250 for 4 h and distained with 30% methanol and 10% acetic acid for 1 h. To explore the effect of inhibitors, the final concentrations of the inhibitors were set as 6 mM PMSF, 6 mM 1,10-phenanthroline and 60 μM E-64, respectively. The mixture of the diluted intestinal extracts and inhibitor solutions was prepared before mixing with sample buffer.

### 2.5. Rheological Properties

An equal volume of the intestinal extract solution, or the solution containing enzyme inhibitors, was combined with the CCF solution. The mixing process lasted for 30 min at pH 9.0 and 40 °C. Then, the rheological properties of CCF treated with intestinal extracts and protease inhibitor were detected by a rheometer (Discovery HR-1, TA Instruments Menu Co., Ltd., New Castle, DE, USA). Flow sweep was performed with a parallel plate diameter of 40 mm at 15 °C. The CCF solution with an equal amount of buffer A was used as a control group.

## 3. Results

### 3.1. Optimal Conditions of Gelatinolytic Enzymes in Sea Cucumber Intestinal Extract

**Figure 1:**
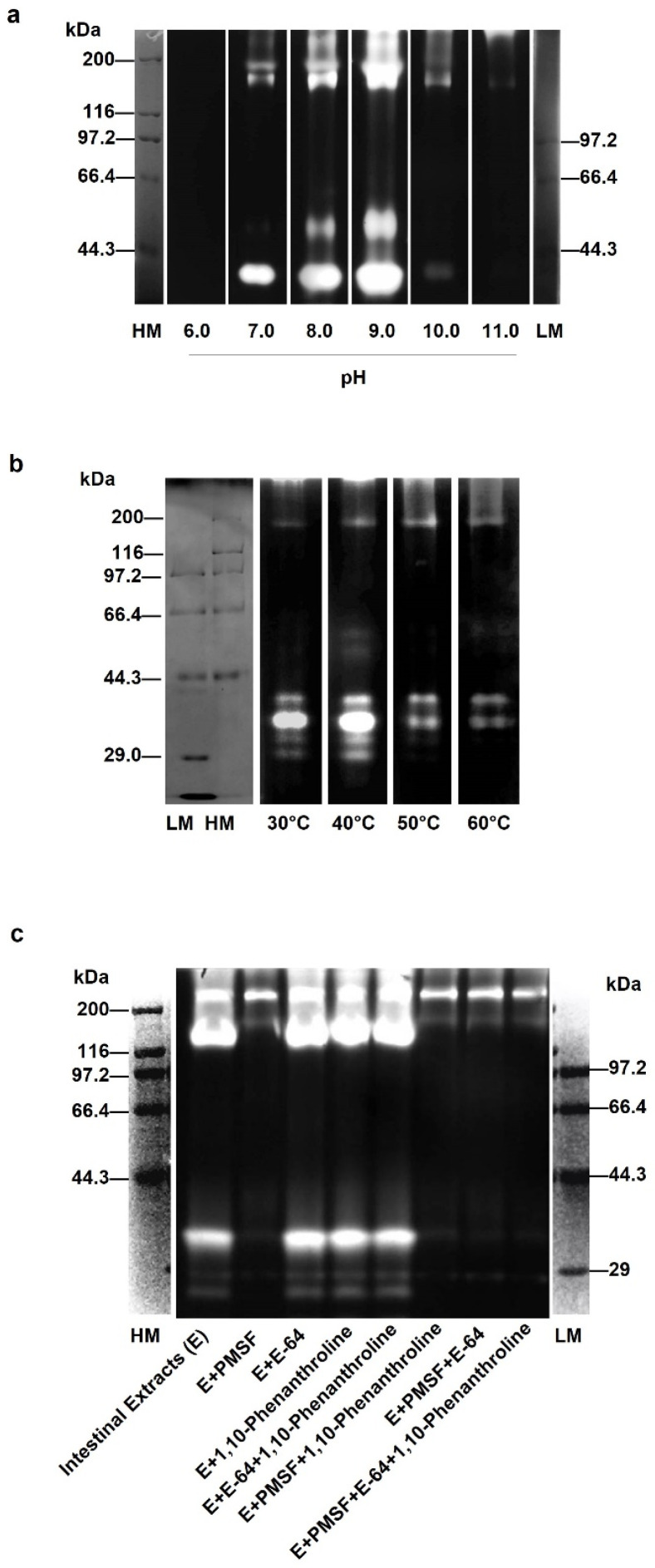
Activity determination of sea cucumber intestinal extracts by gelatin zymography. Effects of different pH (**a**), temperatures (**b**), inhibitors (**c**) on enzymes from sea cucumber intestinal extracts. HM and LM: high and low molecular weight markers; E: sea cucumber intestinal extracts.

In order to explore the optimal conditions of collagen-degrading enzymes in the sea cucumber intestine, gelatin zymography was performed at different pH values and temperatures. As shown in Figure 1a, the gelatinolytic activity of the intestine extract of the sea cucumber was detected at pH values from 6.0 to 11.0. No bands appeared at pH 6.0, but clear bands were observed in the pH range from 7.0 to 9.0, with the brightest bands at pH 9.0. However, when the pH was greater than 9.0, the enzyme activity decreased with increasing pH. Almost no bands were observed until pH 11.0. As shown in Figure 1b, the enzymes in the intestine extract had better activity at 30 °C and 40 °C than those at 50 °C and 60 °C. Among them, the band at 40 °C was significantly brighter than that at 30 °C. These results indicated that the optimal pH and temperature of the endogenous enzymes for gelatinolytic activity in the sea cucumber intestine extract were 9.0 and 40 °C, respectively. Under these conditions, the enzymes degraded more gelatin. In addition, the molecular weight of the endogenous enzyme was mainly distributed in 29.0–44.3 kDa. Weak bands were observed at around 133 kDa. This may be due to polymers or precursors of some enzymes [23].

In order to further clarify the properties of the enzymes in the sea cucumber intestinal extract, a variety of protease inhibitors were used, including 1,10-phenanthroline, E-64 and PMSF. As shown in Figure 1c, compared with the control group without any inhibitors, hardly any bands were observed at about 34 kDa after the addition of PMSF. It was inferred that the activity of the endogenous enzymes of sea cucumber intestine extracts to degrade gelatin was effectively suppressed. However, the change in brightness of the bands with 1,10-phenanthroline and E-64 added, alone or in combination, was not noticeable. It is well known that 1,10-phenanthroline, E-64, and PMSF are inhibitors of metalloproteinases, cysteine proteases, and serine proteases, respectively. It was concluded that the endogenous enzymes of the sea cucumber intestine extracts were inhibited by the serine protease inhibitor, while the cysteine protease and metalloproteinase inhibitor had no obvious inhibitory effects. When three inhibitors were added at the same time, the lane was darkest, indicating that the degradation of gelatin by endogenous enzymes was blocked. Therefore, it is speculated that this endogenous enzyme may be dominated by serine proteases, while containing trace amounts of metalloproteinases and cysteine proteases.

### 3.2. Effect of Sea Cucumber Intestinal Extract on Rheological Properties of CCF

**Figure 2:**
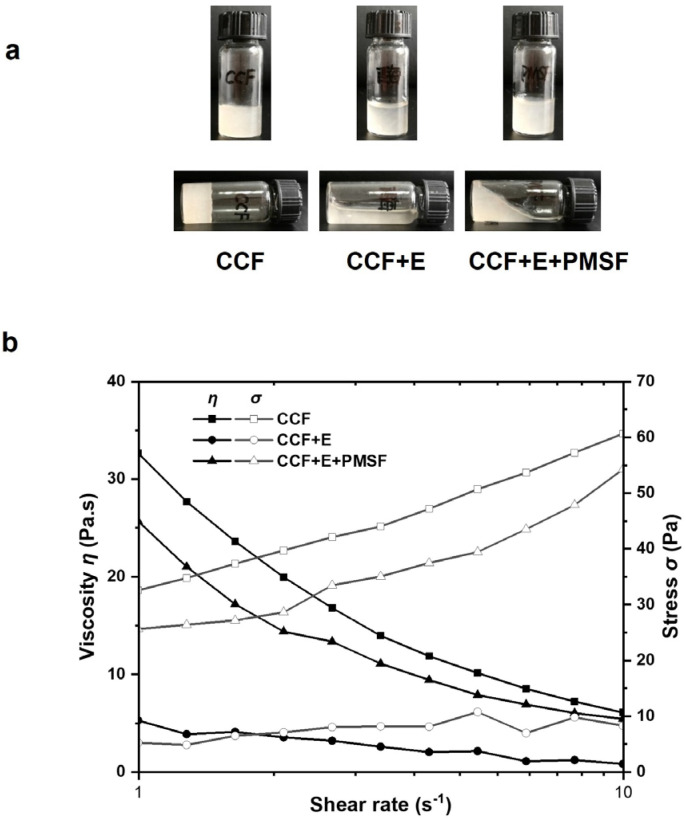
Effects of sea cucumber intestinal extracts on the appearance and rheological properties of sea cucumber crude collagen fibers. (**a**) The apparent morphology of CCF, CCF+E, CCF+E+PMSF. (**b**) Viscosity and stress of samples at different shear rates. CCF: crude collagen fibers; E: sea cucumber intestinal extracts; PMSF: phenylmethylsulfonyl fluoride.

Due to the gel properties of CCF, the collagenolytic activity of intestinal extracts during body wall softening was investigated by in vitro mixing. After adding sea cucumber intestine extract and/or the protease inhibitor PMSF, changes in the morphology of CCF were observed after standing at room temperature. When CCF existed alone, it exhibited a nonflowing solid-like state. CCF changed from a gel state to a flowing state after the addition of intestinal extracts. In addition, with the addition of PMSF, the state of CCF was between solid and liquid (Figure 2a).

Shear rate sweep is a typical model to evaluate rheological behavior. To further determine the flow behavior of the samples, the apparent viscosity (*η*) and shear stress (*σ*) at different shear rates were examined (Figure 2b). When the shear rate was 1 s^−1^, the viscosity of the CCF was 32.7 Pa·s. The viscosity of CCF decreased to 5.3 Pa·s after intestinal extract was added. In the presence of both intestinal extract and the protease inhibitor PMSF, the viscosity of CCF was 25.7 Pa·s, slightly lower than that of the untreated CCF but higher than that of only extract treated CCF. With the increasing shear rate, the viscosity and shear stress of the CCF decreased and increased rapidly, respectively. The CCF samples with both intestinal extract and PMSF also showed a similar trend, but the overall reduction was slightly smaller than that of CCF. These results suggested that CCF possessed a non-Newtonian shear thinning property. However, the viscosity and shear stress values of CCF samples treated with intestinal extract alone were low and did not change significantly. In summary, the endogenous enzymes of the intestine extract reduced the viscosity of CCF by simulating the process of body wall softening in vitro. The serine protease inhibitor PMSF was able to restrain this deterioration to some extent. Combined with the results of gelatin zymography, it is concluded that serine protease in the sea cucumber intestine was involved in the degradation of CCF.

## 4. Discussion

Sea cucumbers are easily impacted by changes in environmental factors, such as salinity changes, temperature rise, and ultraviolet radiation, and softening of the body wall usually occurs [24]. Along with softening, the gut is exhaled from the body, touching and degrading the body wall. Studying the effects of sea cucumber intestinal extracts on CCF is very important for elucidating the mechanism involved in the deterioration of sea cucumbers.

Confirmation of the enzyme property is the primary part for studying its function. Gelatin zymography separates enzymes that can degrade gelatin by their molecular weight and concentrate them in small bands. The gelatin degradation zone shows bright bands because it cannot be stained. The greater the enzyme activity and content, the greater the brightness and width of the band [25]. From the results in Figure 1, it was confirmed that the main endogenous enzymes in the sea cucumber intestine were serine proteases. Their optimal pH and temperature were also obtained at pH 9.0 and 40 °C, respectively, by gelatin zymography. In a previous study, Wu et al. [13] reported a metalloproteinase (MP) from the sea cucumber body wall using the same method. The optimal pH of that MP was 9.0, and the optimal temperature was 40–45 °C. Yan [26] reported that serine proteinase (SP), which can be cloned by cDNA and expressed in vitro, hydrolyzed gelatin well at pH 6.0–9.0 and 35–40 °C. The molecular weight of SP, which can degrade collagen, was about 34 kDa. These findings are similar to our results. In addition, gelatinases A and B, namely matrix metalloproteinases 2 and 9, were reported to be synthesized and secreted in vivo as latent pro-enzymes with molecular weights between 72–225 kDa [23]. Therefore, there might also be some precursor enzymes in the extracts of sea cucumber intestines in this study. These results suggested that a couple of enzymes from sea cucumber intestines participated in the breakdown of collagen from the sea cucumber body wall.

Previous studies have shown that marine collagen is a complex viscous elastomer. Their elastic or viscous modulus increases with frequency under the frequency sweep mode of rheology [27,28]. Dong et al. investigated the elastic modulus *G″* and found that *G′* increased significantly at 45 °C, and stabilized after 65 °C. As a result, the proteins aggregated in an orderly manner, and the sea cucumber paste completed the transformation from viscous protein paste to solid [29]. This corresponds to the solid gel state presented by CCF alone in our results. In addition, other reports indicated that the viscosity of pepsin-solubilized collagen of grass carp and sea cucumber decreased when the temperature increased, which may be due to the reduction in hydroxyproline content and the breakage of covalent or hydrogen bonds due to high temperature [30,31]. In addition, trypsin is the most typical serine endopeptidase. Liu et al. [32] used commercial trypsin to degrade sea cucumber collagen fibers, and only a decrease in the molecular weight of pepsin-solubilized collagen was observed. In our study, insoluble CCF was the main object, and morphology and rheology were effective methods to detect the effect of intestinal extract on CCF. The viscosity of CCF decreased with the shear rate after treatment with the intestinal extract. This may be because intestinal extracts from sea cucumbers disrupt serine residue sites in CCF. The deterioration of the sea cucumber body wall can be attenuated by the addition of the serine protease inhibitor PMSF.

## 5. Conclusions

In this study, it was identified that the enzymes in sea cucumber intestines were mainly serine proteases with a molecular weight of 34 kDa. They were involved in the reduction of the viscosity of sea cucumber CCF. Therefore, the degradation of collagen during the body wall softening process of sea cucumbers can be reduced by inhibiting the activity of serine protease, and then maintaining the quality of sea cucumbers during processing.

## Data Availability

Not applicable.
